# The Importance of General Self-Efficacy for the Quality of Life of Adolescents with Diabetes or Juvenile Rheumatoid Arthritis Over Time: A Longitudinal Study among Adolescents and Parents

**DOI:** 10.3389/fped.2013.00040

**Published:** 2013-11-20

**Authors:** Jane M. Cramm, Mathilde M. H. Strating, Anna P. Nieboer

**Affiliations:** ^1^Institute of Health Policy and Management, Erasmus University, Rotterdam, Netherlands

**Keywords:** self-efficacy, quality of life, chronic illness, adolescent, parent, diabetes, juvenile rheumatoid arthritis

## Abstract

**Purpose:** To (i) investigate the influence of general self-efficacy on quality of life outcomes over time among adolescents with type I diabetes or juvenile rheumatoid arthritis (JRA), (ii) investigate parents’ perceptions of general self-efficacy and quality of life of adolescents with diabetes or JRA over time, and (iii) identify possible differences in the evaluations of adolescents and parents.

**Methods:** This study included adolescents aged 12–25 years with type I diabetes or JRA and their parents. At T1, 171/573 (30% response rate) adolescents with diabetes or JRA and 229/563 (41% response rate) parents completed the questionnaire. At T2, 230/551 (42% response rate) adolescents and 220/559 (39% response rate) parents still participating in the study completed the questionnaire. A total of 112 adolescents and 143 parents filled in the questionnaires at both T1 and T2.

**Results:** Adolescents perceived significant improvement in their general self-efficacy and reduced quality of life over time, whereas parents’ perceptions did not change. According to adolescents and parents, physical functioning was better among adolescents with diabetes than among those with JRA. Regression analyses of adolescents’ data showed that general self-efficacy at T1 (β = 0.13; *p* ≤ 0.10) and changes in general self-efficacy (β = 0.22; *p* ≤ 0.01) predicted quality of life at T2. Parents’ responses revealed that adolescents’ general self-efficacy at T1 (β = 0.16; *p* ≤ 0.05) and changes in adolescents’ general self-efficacy (β = 0.18; *p* ≤ 0.05) predicted adolescents’ quality of life at T2.

**Conclusion:** General self-efficacy and changes therein positively affected quality of life in adolescents with diabetes or JRA over time, as perceived by adolescents and parents. These findings emphasize the need for the implementation of interventions aiming to improve general self-efficacy in these populations.

## Introduction

Chronically ill adolescents such as those with type I diabetes or juvenile rheumatoid arthritis (JRA) must deal with taking medication, engaging in daily physical activity, maintaining a healthy diet, and proper disease management to reduce the negative effects of the disease on their quality of life (1–3).

The most common symptoms of type I diabetes are blurred vision, decreased mental sharpness, extreme thirst and hunger, fatigue, the frequent need to urinate, frequent skin infections, weight loss (despite increased appetite), and slow-healing wounds.

The most common symptoms of all forms of JRA include joint pain and swelling, which may come and go but are most often persistent; joint stiffness in the morning; limping; and unpredictable changes in symptoms from asymptomatic periods (remission) to flare-ups and pain. The long-term effects of JRA include joint contracture and joint damage.

Although JRA and diabetes differ in their origin and symptoms, both conditions require constant monitoring to avoid complications and effective disease management (e.g., treatment compliance, healthy diet, taking medication, pain management, physical activities) is similar for adolescents with these conditions (4). Investigating and comparing their quality of life could increase our understanding of how different diseases may or may not differentially affect quality of life (5).

Self-efficacy is important for effective disease management. Research has shown that self-efficacy predicts adherence (taking medication), health behavior (physical activities), effective pain management, and disease management (6–14), which are expected to be important for quality of life. While diabetes and JRA are conditions with unique aspects, they also have comparable self-efficacy tasks, such as managing symptoms and treatment, forming relationships with care providers, maintaining a positive self-image, and preparing for an uncertain future (4). Use of a generic self-efficacy measure instead of a disease specific self-efficacy measure enables the comparison of adolescents with diabetes and those with JRA, which may be useful in predicting health behaviors and quality of life across conditions. General self-efficacy refers to the belief in one’s competence to attempt difficult or novel tasks, and to cope with adversity arising from specific demanding situations (15–18). When setbacks occur, adolescents with higher levels of general self-efficacy may recover more quickly and remain committed to their goals. As such, general self-efficacy is considered to be an important factor in coping with the challenges and demands presented by these chronic conditions during adolescence (1).

Longitudinal studies showed that self-efficacy predicts quality of life among patients with multiple sclerosis (19), myocardial infarction (20), cancer (21, 22), arthritis (23), and Chronic Obstructive Pulmonary Disease (24). Cramm and colleagues (1) also found a cross-sectional relationship between general self-efficacy of adolescents with a variety of chronic conditions and their quality of life, but a lack of longitudinal studies investigating if this relationship among adolescents with JRA or diabetes over time persists. Since earlier longitudinal studies showed that self-efficacy predicted quality of life among patients with other chronic conditions the hypothesis of this study is that self-efficacy also predicts quality of life among adolescents with diabetes and JRA. Furthermore, it remains unknown whether this relationship differs across conditions. The first aim of this study is to investigate the influence of general self-efficacy on quality of life outcomes over time among adolescents with diabetes or JRA. Since self-efficacy is thought of to be modifiable through intervention, it may be used to improve quality of life of chronically ill adolescents.

Adolescent and proxy reports from parents are often used in pediatric and adolescent care and discrepancies between adolescents’ and parents’ perceptions of adolescents’ quality of life have been identified, but research comparing the longitudinal relationship between adolescents and proxy reports from parents of changes in adolescents’ general self-efficacy skills and quality of life over time is lacking. Therefore, the second aim of this study is to investigate parents’ perceptions of general self-efficacy and quality of life of adolescents with diabetes or JRA over time. Studying the effects of general self-efficacy and changes therein on quality of life among adolescents with diabetes or JRA, as assessed by adolescents and parents over time allows us to compare their findings and identify possible differences in their evaluations (aim three).

## Materials and Methods

This longitudinal study surveyed adolescents with type I diabetes or JRA and their parents at two measurement points (T1 in 2010 and T2 in 2011). Mean time between T1 and T2 was 16.7 ± 1.5 (range 15–21) months. Eligible participants were 12–25-year-olds with diabetes or JRA in active pediatric follow-up (at least one contact moment in the past year) at participating hospital departments. These departments joined a quality improvement program called On Your Own Feet Ahead (1, 25). The aim of this program is to improve care for adolescents with diabetes or JRA in seven hospital departments in The Netherlands.

Approval for the study was obtained from the Erasmus Medical Center Institutional Review Board. Eligible adolescents and parents received written information and a unique access code, and were invited to complete an online questionnaire. Non-respondents received a postal reminder after 2 weeks, including a printed copy of the questionnaire. At T1, 171 out of a total of 573 adolescents with diabetes or JRA (30% response rate) filled in the questionnaire and 229 out of a total of 563 parents (41% response rate) completed the questionnaire. At T2 all patients (and their parents) still included in the study were asked again to fill in the questionnaire. At T2, 230 out of 551 adolescents (42% response rate) and 220 out of 559 parents (39% response rate) completed the questionnaire. A total of 112 adolescents and 143 parents filled in the questionnaires at both T1 and T2.

### Measures

The 10-item general self-efficacy scale was used to assess adolescents’ and parents’ perceptions of self-efficacy in adolescents with diabetes or JRA (5). The ten items are: “I can always manage to solve difficult problems if I try hard enough,” “if someone opposes me, I can find the means and ways to get what I want,” “it is easy for me to stick to my aims and accomplish my goals,” “I am confident that I could deal efficiently with unexpected events,” “thanks to my resourcefulness, I know how to handle unforeseen situations,” “I can solve most problems if I invest the necessary effort,” “I can remain calm when facing difficulties because I can rely on my coping abilities,” “when I am confronted with a problem, I can usually find several solutions,” “if I am in trouble, I can usually think of a solution,” and “I can usually handle whatever comes my way.” Respondents were asked to rate their agreement with items on a four-point scale ranging from 1 (“not at all true”) to 4 (“exactly true”). No time frame was provided in asking about perceptions of self-efficacy. Parents were asked to rate their agreement on how these items apply to their child using the same four-point scale. A total score (10–40) was obtained by summing the responses to each of the 10-items. Cronbach’s alpha values of the general self-efficacy scale among adolescents at T1 (0.83) and T2 (0.88) and parents at T1 (0.93) and T2 (0.92) indicated excellent reliability.

The instrument has been widely used among adolescents, patients, and parents [e.g., Ref. (1, 26–29)] Evidence of the validity and predictive nature of the general self-efficacy scale has been previously published [e g., Ref. (30–35)]. These studies reported strong negative associations of general self-efficacy with, for example, depression, anxiety, stress, and burn-out and strong positive relationships were found with optimism.

Adolescents’ and parents’ perceptions of quality of life of adolescents with diabetes or JRA were assessed with the 37-item DISABKIDS condition-generic module questionnaire (2, 3). The DISABKIDS was specifically developed to assess quality of life among chronically ill children and adolescents (36). The DISABKIDS consists of four versions: a self-report version for children, a proxy version for parents and a child, and proxy version for those younger than 8 years (The DISABKIDS – Smileys measure). The 37-item DISABKIDS condition-generic module questionnaire which we used in our study contains six dimensions (independence, physical limitation, emotion, social exclusion, social inclusion, and treatment). Psychometric tests revealed that this instrument proved satisfactory internal consistency for all dimensions (36, 37) and showed satisfying results regarding the instrument’s construct validity, convergent and discriminant validity (37, 38). Responses to each item are structured using a five-point Likert scale ranging from 1 (“never”) to 5 (“always”). Following the method of the developers of the instrument overall quality of life scores were transformed linearly to a 0–100 scale, with 100 indicating the highest quality of life. Cronbach’s alpha values of the DISABKIDS condition-generic module among adolescents at T1 (0.82) and T2 (0.84) and parents at T1 (0.83) and T2 (0.85) indicated good internal reliability among our study sample.

The questionnaire further asked respondents to provide information about background variables, such as age and gender. In addition, to account for the severity of chronic conditions, we used the SF-20 physical functioning scale to assess adolescents’ and parents’ perceptions of physical functioning among adolescents with diabetes or JRA (39, 40). Physical functioning is assessed by limitations due to health in a variety of physical activities, ranging from strenuous to basic. Responses to each item are structured using a three-point Likert scale. The construction of this measure is described by Stewart and colleagues (41). Briefly, the SF-20 physical functioning scale obtains a score by summing the responses. The scores are reversed so that a high value indicates better physical functioning and is transformed linearly to range from 0 to 100, with higher scores indicating higher levels of physical functioning. These transformations are based on developer instruction. Support for the reliability and construct validity of the SF-20 is provided in previously published documents [e.g., Ref. (41, 42)].

### Statistical analyses

Descriptive analyses included the calculation of means and standard deviations. Two-tailed, paired *t*-tests were used to investigate improvements in quality of life and general self-efficacy over time (T2–T1), as perceived by adolescents and parents. Differences in quality of life, general self-efficacy, age, gender, and physical functioning between adolescents with diabetes and those with JRA were established using chi-squared and two-tailed, independent sample *t*-tests. Next, we performed correlation analyses to identify significant relationships between background characteristics, self-efficacy at T1, and changes in self-efficacy and quality of life, as perceived by adolescents and parents. Point biserial correlation analyses were used to investigate the relationship between the dichotomous independent variables and quality of life. Significant variables in the univariate analyses were included in the multivariate analyses. Multiple regression analyses (using listwise deletion of missing cases) were then performed using data from adolescents and parents separately to determine whether general self-efficacy at T1 and/or changes in general self-efficacy predicted quality of life at T2, after controlling for significant background variables and quality of life at T1. We analyzed parents and adolescents separately (not as dyads). All statistical analyses were conducted with SPSS software (version 20.0; SPSS IBM).

## Results

About half (54.5%) of the adolescents were female and their mean age was 16.0 ± 2.3 (range 12–22) years. The majority (66.4%) of the respondents reported having diabetes and 33.6% had JRA. The majority (86%) of participating parents were mothers, and 14% were fathers. Parents’ mean age was 47 ± 4.9 (range 36–66) years. Paired *t*-test results showed that adolescents perceived significant improvement in their general self-efficacy and reduced quality of life over time, whereas parents’ perceptions did not change (Table 1).

**Table 1:** **Adolescents and parents’ perceptions of changes in quality of life and general self-efficacy over time**.

	*N*	Baseline assessment (T1, 2010)		Follow-up assessment (T2, 2011)		T2–T1		
		M	SD	M	SD	M	SD	*p*^a^
**ADOLESCENTS’ PERCEPTIONS**								
Quality of life	110	64.58	(11.32)	62.75	(12.04)	−1.84	(9.29)	<0.05
General self-efficacy	112	29.34	(4.19)	30.76	(5.14)	1.42	(4.31)	<0.001
**PARENTS’ PERCEPTIONS**								
Quality of life	142	62.67	(10.58)	62.86	(12.19)	0.19	(7.99)	0.778
General self-efficacy	142	30.39	(5.74)	30.95	(5.38)	0.56	(4.95)	0.186

*M, mean; SD, standard deviation*.

*^a^ Paired *t*-test, T1 vs. T2*.

Table 2 displays differences in quality of life, age, gender, physical functioning, and general self-efficacy between adolescents with diabetes and those with JRA, as perceived by adolescents. These results show that the level of physical functioning was significantly higher among adolescents with diabetes than among those with JRA (87.0 vs. 69.0, *p* ≤ 0.001). No difference was found between groups in quality of life (T1 or T2), age, gender, general self-efficacy at T1, or changes in general self-efficacy (T2–T1).

**Table 2:** **Background characteristics and perceived quality of life and general self-efficacy among adolescents with diabetes or juvenile rheumatoid arthritis**.

		Total	JRA	Diabetes	χ^2^	*t*	*p*
Quality of life at T1 (2010)	*n*	127	54	73		1.368	0.174
	Mean	65.3	67.0	64.1	
	SD	11.8	12.8	11.0	
Quality of life at T2 (2011)	*n*	156	83	73		−0.009	0.993
	Mean	62.4	62.4	62.4	
	SD	11.4	9.9	12.9	
Age (years) at T1	*n*	127	54	73		0.087	0.931
	Mean	16.0	16.0	15.9	
	SD	2.2	2.6	1.9	
Gender (female) at T1	*n*	127	54	73	0.312		0.591
	%	58.3	61.1	56.2	
Physical functioning (SF-20) at T1	*n*	123	51	72		−3.718	≤0.001
	Mean	79.5	69.0	87.0	
	SD	26.5	30.0	20.8	
General self-efficacy at T1	*n*	127	54	73		1.089	0.278
	Mean	29.4	29.9	29.1	
	SD	4.2	4.7	3.8	
Changes in general self-efficacy (T2–T1)	*n*	112	39	73		−1.642	0.103
	Mean	1.4	0.5	1.9	
	SD	4.3	5.0	3.8	

JRA, juvenile rheumatoid arthritis; SD, standard deviation.

*Results are based on respondents who filled in questionnaires at both T1 and T2 only*.

Table 3 presents differences in quality of life, age, gender, physical functioning, and general self-efficacy between adolescents with diabetes and those with JRA, as perceived by parents. According to the parents, physical functioning levels were also significantly higher among adolescents with diabetes than among those with JRA (93.0 vs. 66.2, *p* ≤ 0.001). Furthermore, parents perceived a greater degree of improvement in general self-efficacy among adolescents with JRA than among those with diabetes (1.7 vs. −0.1, *p* ≤ 0.05). No difference was found between groups in parents’ perceptions of adolescents’ quality of life (T1 or T2), parents’ age or gender, or parents’ perceptions of adolescents’ general self-efficacy at T1.

**Table 3:** **Parents’ background characteristics and perceived quality of life and general self-efficacy of adolescents with diabetes or juvenile rheumatoid arthritis**.

		Total	JRA	Diabetes	χ^2^	*t*	*p*
Parents’ perceptions of adolescents’ quality of life at T1 (2010)	*n*	169	77	92		0.589	0.556
	Mean	63.2	63.7	62.7			
	SD	11.0	10.2	11.6			
Parents’ perception of adolescents’ quality of life at T2 (2011)	*n*	169	76	93		−0.821	0.413
	Mean	62.4	61.6	63.1	
	SD	11.8	10.0	13.1	
Age of parents (years) at T1	*n*	161	72	89		−1.017	0.311
	Mean	46.9	46.5	47.2	
	SD	4.8	5.3	4.4	
Gender of parents (female) at T1	*n*	165	75	147	0.045		1.000
	%	87.3	86.7	87.8	
Parents’ perceptions of adolescents’ physical functioning (SF-20) at T1	*n*	169	76	93		−6.457	≤0.001
	Mean	81.0	66.2	93.0	
	SD	28.3	33.7	14.6	
Parents’ perceptions of adolescents’ general self-efficacy at T1	*n*	169	77	92		−0.335	0.738
	Mean	30.4	30.2	30.5	
	SD	5.9	6.4	5.4	
Parents’ perceptions of changes in adolescents’ general self-efficacy (T2–T1)	*n*	142	50	92		2.022	≤0.05
	Mean	0.6	1.7	−0.1	
	SD	5.0	4.3	5.2	

JRA, juvenile rheumatoid arthritis; sd, standard deviation.

*Results are based on respondents who filled in questionnaires at both T1 and T2 only*.

Univariate analyses of adolescents’ perceptions showed that quality of life at T1 (*p* ≤ 0.001), physical functioning (*p* ≤ 0.05), general self-efficacy at T1 (*p* ≤ 0.01), and changes in general self-efficacy (*p* ≤ 0.05) were all positively associated with quality of life at T2 (Table 4). A negative relationship was found between female gender and quality of life at T2 (*p* ≤ 0.01). No significant relationship was found between quality of life at T2 and adolescents’ age or type of chronic condition.

**Table 4:** **Correlations with quality of life in adolescents with diabetes or juvenile rheumatoid arthritis at T2 (2011): adolescents’ perceptions**.

	Quality of life at T2 (2011) (*n* = 110)	
	*r*	*t*^a^
Quality of life at T1 (2010)	0.69***	
Age (years) at T1	0.08	
Gender (female) at T1		−2.345**
Type of chronic condition (JRA) at T1		0.644
Physical functioning (SF-20) at T1	0.18*	
General self-efficacy at T1	0.22**	
Changes in general self-efficacy (T2–T1)	0.20*	

***p ≤ 0.001; **p ≤ 0.01; *p ≤ 0.05 (one-sided).

*JRA, juvenile rheumatoid arthritis*.

*^a^ Point biserial correlations*.

Correlation analyses of parents’ perceptions showed that adolescents’ quality of life at T1 (*p* ≤ 0.001), adolescents’ general self-efficacy at T1 (*p* ≤ 0.05), and changes in adolescents’ general self-efficacy (T2–T1, *p* ≤ 0.01) were positively related to parents’ perceptions of adolescents’ quality of life at T2 (Table 5). A negative relationship was found between female gender of adolescents, female gender of parents and quality of life at T2 (both *p* ≤ 0.01). No significant relationship was found between quality of life at T2 and parents’ or adolescents’ age, type of chronic condition, or physical functioning.

**Table 5:** **Correlations with quality of life in adolescents with diabetes or juvenile rheumatoid arthritis at T2 (2011): parents’ perceptions**.

	Parents’ perceptions of adolescents’ quality of life at T2 (2011) (*n* = 107)	
	
	*r*	*t*^a^
Parents’ perception of adolescents’ quality of life at T1 (2010)	0.76***	
Age of parents (years) at T1	0.06	
Age of adolescents (years) at T1	0.08	
Gender of adolescents (female) at T1		−2.325**
Gender of parents (female) at T1		2.191**
Type of chronic condition (JRA) at T1		0.751
Parents’ perceptions of adolescents’ physical functioning (SF-20) at T1	0.09	
Parents’ perceptions of adolescents’ general self-efficacy at T1	0.15*	
Parents’ perceptions of changes in adolescents’ general self-efficacy (T2–T1)	0.20**	

***p ≤ 0.001; **p ≤ 0.01; *p ≤ 0.05 (one-sided).

*JRA, juvenile rheumatoid arthritis*.

*^a^ Point biserial correlations*.

Table 6 displays adolescents’ perceptions of quality of life predictors among adolescents with diabetes or JRA at T2, as assessed by multiple regression analyses. After controlling for quality of life at T1, gender, and physical functioning, general self-efficacy at T1 (β = 0.13, *p* ≤ 0.05) and changes in general self-efficacy (β = 0.22, *p* ≤ 0.001) predicted quality of life at T2. The multiple regression model explained 52.3% of variance.

**Table 6:** **Quality of life predictors in adolescents with diabetes or juvenile rheumatoid arthritis at T2 (2011; multiple regression): adolescents’ perceptions**.

	Quality of life at T2 (2011) (*n* = 107)
Quality of life at T1 (2010)	0.66***
Gender (female) at T1	−0.13*
Physical functioning (SF-20) at T1	−0.10
General self-efficacy at T1	0.13*
Changes in general self-efficacy (T2–T1)	0.22***
Adjusted *R*^2^	52.3%
*F*	24.458

***p ≤ 0.001; *p ≤ 0.05 (one-sided).

Parents’ perceptions of quality of life predictors for adolescents with JRA or diabetes are displayed in Table 7. After controlling for quality of life at T1 and parents’ and adolescents’ genders, general self-efficacy at T1 (β = 0.16, *p* ≤ 0.05) and changes in general self-efficacy (β = 0.18, *p* ≤ 0.05) predicted adolescents’ quality of life at T2. The multiple regression model explained 59.5% of variance.

**Table 7:** **Quality of life predictors in adolescents with diabetes or juvenile rheumatoid arthritis at T2 (2011; multiple regression): parents’ perceptions**.

	Parents’ perceptions of adolescents’ quality of life at T2 (2011) (*n* = 105)
Parents’ perception of adolescents’ quality of life at T1 (2010)	0.67***
Gender of adolescents (female) at T1	−0.06
Gender of parents (female) at T1	−0.09
Parents’ perceptions of adolescents’ general self-efficacy at T1	0.16*
Parents’ perceptions of changes in adolescents’ general self-efficacy (T2–T1)	0.18*
Adjusted *R*^2^	59.5%
*F*	26.571

***p ≤ 0.001; *p ≤ 0.05 (one-sided).

## Discussion

Purpose of this study was to (i) investigate the influence of general self-efficacy on quality of life outcomes over time among adolescents with type I diabetes or JRA, (ii) to investigate parents’ perceptions of general self-efficacy and quality of life of adolescents with diabetes or JRA over time, and (iii) identify possible differences in the evaluations of adolescents and parents.

The mean general self-efficacy scale scores for adolescents with diabetes or JRA (29.4) and their parents (30.4) in this study sample were comparable to those among average students without (known) health conditions in various countries (range 29.4–33.4) (18). The adolescents in this study were equally confident in their abilities to deal with demanding and difficult situations as are their peers, despite having diabetes or JRA. Our findings confirmed our expectations that general self-efficacy and changes therein would affect quality of life in these adolescents.

We found significant univariate and multivariate relationships between general self-efficacy and changes therein on quality of life at T2, even after controlling for quality of life at T1, age, gender, and type of chronic condition. We also found evidence for a longitudinal relationship between changes in general self-efficacy and quality of life of chronically ill adolescents, according to adolescents’ and parents’ perspectives. These findings emphasize the importance of attention to general self-efficacy skills among adolescents with diabetes or JRA.

An interesting difference found in the longitudinal perspectives of adolescents and parents is that while adolescents perceived general self-efficacy to improve and quality of life to decline over time, parents’ perceptions of their children’s general efficacy and quality of life did not change over time. A reduction in quality of life over time has also been identified in adolescents with physical disabilities (43) and “healthy” adolescents (44). This decrease may be explained by the important life transitions through which these individuals pass as they enter adulthood. Furthermore, in a longitudinal study of healthy adolescents’ and parents’ perceptions of adolescents’ quality of life, Jozefiak and colleagues (44) also found that adolescents reported a reduction in quality of life while parents perceived no significant change. These results indicate that self-reports and proxy reports from parents are not interchangeable. Parents may not be aware of changes experienced by chronically ill adolescents, or their effects on general self-efficacy and quality of life. A focus on adolescents’ as well as parents’ perceptions is therefore recommended.

In addition to investigating the longitudinal relationship between general self-efficacy and quality of life, we also investigated differences in quality of life, age, gender, physical functioning, and general self-efficacy between adolescents with diabetes and those with JRA, as perceived by adolescents and parents. The results revealed a significant difference in the level of physical functioning; adolescents with diabetes and their parents reported much higher levels of physical functioning than did those with JRA. Adolescents’ perceived quality of life, self-efficacy, and changes in general self-efficacy did not differ between chronic conditions.

The most important limitation of our study was the low response rate at T1 and T2; non-response bias may have influenced our study findings. However, these rates are similar to those reported in other studies in which respondents received questionnaires by mail (45, 46). The major strength of our study is that we identified a longitudinal relationship between changes in self-efficacy and quality of life over time, according to adolescents and parents.

## Conclusion

General self-efficacy and changes therein positively affected the quality of life of adolescents with diabetes or JRA over time, as perceived by adolescents and parents. Self-efficacy might be an important target of interventions for improving quality of life of chronically ill adolescents. These findings are important for professionals and health care organizations aiming to improve quality of life in adolescents with diabetes or JRA, and emphasize the need for the implementation of interventions aiming to improve general self-efficacy in these populations.

## Conflict of Interest Statement

The authors declare that the research was conducted in the absence of any commercial or financial relationships that could be construed as a potential conflict of interest.
